# Insights on the differentiation of stillbirths and early neonatal deaths: A study from the Child Health and Mortality Prevention Surveillance (CHAMPS) network

**DOI:** 10.1371/journal.pone.0271662

**Published:** 2022-07-21

**Authors:** Elizabeth Quincer, Rebecca Philipsborn, Diane Morof, Navit T. Salzberg, Pio Vitorino, Sara Ajanovic, Dickens Onyango, Ikechukwu Ogbuanu, Nega Assefa, Samba O. Sow, Portia Mutevedzi, Shams El Arifeen, Beth A. Tippet Barr, J. Anthony G. Scott, Inacio Mandomando, Karen L. Kotloff, Amara Jambai, Victor Akelo, Carrie Jo Cain, Atique Iqbal Chowdhury, Tadesse Gure, Kitiezo Aggrey Igunza, Farzana Islam, Adama Mamby Keita, Lola Madrid, Sana Mahtab, Ashka Mehta, Paul K. Mitei, Constance Ntuli, Julius Ojulong, Afruna Rahman, Solomon Samura, Diakaridia Sidibe, Bukiwe Nana Thwala, Rosauro Varo, Shabir A. Madhi, Quique Bassat, Emily S. Gurley, Dianna M. Blau, Cynthia G. Whitney

**Affiliations:** 1 Department of Pediatrics and Children’s Healthcare of Atlanta, Emory University, Atlanta, Georgia, United States of America; 2 Emory Global Health Institute, Emory University, Atlanta, Georgia, United States of America; 3 Division of Global HIV and Tuberculosis, Centers for Disease Control and Prevention, Durban, South Africa; 4 Public Health Informatics, The Task Force for Global Health, Atlanta, Georgia, United States of America; 5 Centro de Investigação em Saúde de Manhiça [CISM], Manhica, Mozambique; 6 Kisumu County Department of Health, Kisumu, Kenya; 7 Crown Agents, Freetown, Sierra Leone; 8 College of Health Medical Sciences, Haramaya University, Harar, Ethiopia; 9 Centre pour le Développement des Vaccines (CVD-Mali), Bamako, Mali; 10 MRC Vaccines and Infectious Diseases Analytics (VIDA) Research Unit, Chris Hani Baragwanath Academic Hospital, Soweto, South Africa; 11 International Center for Diarrhoeal Diseases Research (icddr,b), Dhaka, Bangladesh; 12 Center for Global Health, Centers for Disease Control and Prevention, Kisumu, Kenya; 13 Department of Infectious Disease Epidemiology, London School of Hygiene & Tropical Medicine, London, United Kingdom; 14 Instituto Nacional de Saúde [INS], Maputo, Mozambique; 15 Department of Pediatrics, Center for Vaccine Development and Global Health, University of Maryland School of Medicine, Baltimore, Maryland, United States of America; 16 Ministry of Health and Sanitation, Freetown, Sierra Leone; 17 World Hope International, Makeni, Sierra Leone; 18 Kenya Medical Research Institute (KEMRI), Nairobi, Kenya; 19 Kisumu East District Hospital, Kisumu, Kenya; 20 ICAP–Columba University, Makeni, Sierra Leone; 21 Wits Health Consortium, University of Witwatersrand, Johannesburg, South Africa; 22 ISGlobal- Hospital Clinic—Universitat de Barcelona, Barcelona, Spain; 23 Catalan Institution for Research and Advanced Studies (ICREA), Barcelona, Spain; 24 Pediatric Infectious Diseases Unit, Pediatrics Department, Hospital de Sant Joan de Deu, University of Barcelona, Barcelona, Spain; 25 Consorcio de Investigacion Biomedica en Red de Epidemiologia y Salud, Madrid, Spain; 26 Department of Epidemiology, Johns Hopkins Bloomberg School of Public Health, Baltimore, Maryland, United States of America; 27 Center for Global Health, Centers for Disease Control and Prevention, Atlanta, Georgia, United States of America; Federal University of Sergipe, BRAZIL

## Abstract

**Introduction:**

The high burden of stillbirths and neonatal deaths is driving global initiatives to improve birth outcomes. Discerning stillbirths from neonatal deaths can be difficult in some settings, yet this distinction is critical for understanding causes of perinatal deaths and improving resuscitation practices for live born babies.

**Methods:**

We evaluated data from the Child Health and Mortality Prevention Surveillance (CHAMPS) network to compare the accuracy of determining stillbirths versus neonatal deaths from different data sources and to evaluate evidence of resuscitation at delivery in accordance with World Health Organization (WHO) guidelines. CHAMPS works to identify causes of stillbirth and death in children <5 years of age in Bangladesh and 6 countries in sub-Saharan Africa. Using CHAMPS data, we compared the final classification of a case as a stillbirth or neonatal death as certified by the CHAMPS Determining Cause of Death (DeCoDe) panel to both the initial report of the case by the family member or healthcare worker at CHAMPS enrollment and the birth outcome as stillbirth or livebirth documented in the maternal health record.

**Results:**

Of 1967 deaths ultimately classified as stillbirth, only 28 (1.4%) were initially reported as livebirths. Of 845 cases classified as very early neonatal death, 33 (4%) were initially reported as stillbirth. Of 367 cases with post-mortem examination showing delivery weight >1000g and no maceration, the maternal clinical record documented that resuscitation was not performed in 161 cases (44%), performed in 14 (3%), and unknown or data missing for 192 (52%).

**Conclusion:**

This analysis found that CHAMPS cases assigned as stillbirth or neonatal death after DeCoDe expert panel review were generally consistent with the initial report of the case as a stillbirth or neonatal death. Our findings suggest that more frequent use of resuscitation at delivery and improvements in documentation around events at birth could help improve perinatal outcomes.

## Background

Child mortality disproportionately occurs in the perinatal period. In 2018, an estimated 5.2 million deaths occurred worldwide in children under the age of 5 years, with 2.4 million occurring in the first month of life [1]. Of these neonatal deaths, approximately one-third occur in the first day of life. Prevention of perinatal mortality, defined as stillbirths and neonatal deaths within the first 7 days of life, is increasingly important for reducing global child mortality rates [2]. Accurate cause of death data have been shown to improve health outcomes in high-income countries [3, 4]. Obtaining quality data in low- and middle-income countries is hampered by insufficient diagnostics, reporting, resources, and infrastructure [5, 6]. These limitations pose particular challenges for systems that classify and investigate the causes of perinatal death. Historically, researchers have relied upon sparse data not only to assign the cause of death but also to interpret the timing of the event and the relative contribution of maternal factors to the adverse perinatal outcome [7]. While some risk factors for stillbirth and neonatal death overlap, others are distinct and addressed by different sets of interventions. Differentiating early neonatal deaths from stillbirths is an important step toward understanding and preventing mortality in these groups [8, 9].

Studies have found that 1 in 5 neonatal deaths may be misclassified as a stillbirth in low resource settings [10]. Traditional understanding of stillbirth and neonatal death differs across cultures and communities [11]. Birth attendant practices such as avoiding showing a stillborn infant to the mother, the perceived value and social recognition of a stillbirth, and a family’s willingness to discuss stillbirths may all contribute to misclassification of stillbirths and neonatal deaths in surveillance programs [12]. Cultural definitions and practices that differ from the biomedical definitions and expectations around stillbirths and neonatal deaths also pose challenges for classification of these outcomes [13, 14].

The burden of stillbirths and neonatal deaths has led to global initiatives focused on improving birth outcomes and survival in these groups [15]. The World Health Organization (WHO) recommends resuscitation for all newborn infants who do not breathe spontaneously after drying and appropriate stimulation within one minute of birth [16]. Implementation of this guidance is not universal, and maternal access to basic prenatal care, emergency obstetric care, and skilled birth attendants is variable. Training programs in basic resuscitation measures for birth attendants have substantially reduced fresh stillbirths and neonatal deaths in the first 24 hours of life. Evidence from programs like Helping Babies Breathe that targeted interventions improve perinatal outcomes underscores the importance of correct classification and accurate understanding of factors contributing to stillbirth and neonatal death [17–20].

The Child Health and Mortality Prevention Surveillance (CHAMPS) Network aims to better characterize child mortality and improve accuracy of cause of death determination by using minimally invasive tissue sampling (MITS), laboratory diagnostics, verbal autopsy (VA), and available clinical and demographic data in sites in sub-Saharan Africa and South Asia with high rates of under 5 mortality [21]. CHAMPS’ methods assemble more data sources than previously available in perinatal cause of death surveillance programs in high mortality settings and provide an opportunity to improve accuracy of perinatal cause of death classification. This study analyzes perinatal cause of death classification in the CHAMPS network in order to (1) compare indicators of stillbirth and neonatal death from different data sources and (2) evaluate evidence of resuscitation at delivery for stillbirths in accordance with WHO guidelines.

## Methods

### Study population

Promoted by the Bill & Melinda Gates Foundation, the CHAMPS Network currently includes the following sites: Baliakandi/Faridpur, Bangladesh; Harar/Kersa, Ethiopia; Kisumu/Siaya, Kenya; Bamako, Mali; Manhiça, Mozambique; Makeni, Sierra Leone; and Soweto, South Africa. These sites represent geographically and culturally distinct regions with high rates of child mortality and limited available data on disease burden and cause of death as well as strong engagement and partnerships with local and national public health leaders. The study population for this analysis includes early neonatal deaths (<7 days), very early neonatal deaths (<24 hours) and stillbirths enrolled in CHAMPS from December 2016 through January 27, 2021 including MITS and non-MITS enrolled cases (Box 1).

**Box 1. Child Health and Morality Prevention Surveillance (CHAMPS) network case definitions and perinatal mortality terminology.**

**Table:** 

Perinatal Mortality	The number of stillbirths and deaths in neonates in the first 7 days of life
Very early neonatal death	Death in the first 24 hours of life
Early neonate	Death between 24 hours and 7 days of life
Stillbirth	A baby delivered with no signs of life after 28 weeks of gestation
Fresh stillbirth	Stillbirth without skin peeling or sloughing that indicates increased time since in utero demise

### Ethics and IRB approval

Ethics committees overseeing investigators at each site and at Emory University (Atlanta, GA, USA) approved overall and site-specific protocols and this study was approved by the Emory Institutional Review Board (IRB). Parents or guardians of stillborn fetuses or deceased children provided written informed consent before collection of data, specimens, or information on the mothers. All cases were anonymized prior to review. Our de-identified CHAMPS data can be found in the CHAMPS Dataverse [URL to be included upon acceptance].

### Procedures

When a child death or stillbirth occurs in a CHAMPS catchment area, CHAMPS staff are notified by family members or healthcare workers per previously published protocols [21]. In cases of perinatal mortality, if the mother of the stillbirth or neonatal death is a usual resident of the catchment area, the case is eligible for enrollment in CHAMPS. A team of trained staff confirms eligibility for CHAMPS and approaches the parents or guardians of the neonate or stillbirth for consent for MITS procedure, clinical record abstraction and VA. If written and informed consent is obtained and the case is reported to CHAMPS within 24–36 hours (or within 72 hours if refrigeration is used) and the body is available for MITS procedure, the case is eligible for MITS. Non-MITS eligible cases are also enrolled in CHAMPS after written and informed consent. At enrollment, CHAMPS team members collect basic information including if the death was a stillbirth. While non-MITS cases will not have tissue sampling performed, in both MITS and non-MITS enrolled cases clinical record data is abstracted from the maternal health record (in stillbirths and neonatal deaths) and the neonate’s health record (in neonatal deaths) and VA is obtained. These collective data are reviewed by local experts through each site’s determining cause of death (DeCoDe) panels to determine the cause of death for each CHAMPS case, following WHO guidelines for death certification and as previously published [22]. As a part of their review, this expert panel considers all available data to ultimately classify a case as stillbirth or neonatal death.

### Statistical analysis

#### Perinatal classification

A descriptive review of study cases was conducted to characterize available data on indicators of stillbirth and neonatal death from different CHAMPS data sources as well as available data on resuscitation practices at birth. The outcome, or final classification of a case as a stillbirth or neonatal death as certified by the DeCoDe panel was compared to both the initial report of the case by the family member or healthcare worker at CHAMPS enrollment and the documented birth outcome as stillbirth or livebirth from the maternal health record.

For stillbirths and deaths in the first 24 hours of life that occurred in facilities (and for which signs of life could have been assessed at delivery and documented in the clinical record), indicators of signs of life from the clinical record were analyzed and compared with those from the VA. CHAMPS sources queried included: the maternal health record and the VA for stillbirths, and the maternal health record, the child health record, and the VA for neonatal deaths. Signs of life variables from the maternal health record abstraction included in this analysis were heartbeat at birth, breathing or crying at birth, and infant movement at birth. Variables from the child health record abstraction included in this analysis were heartbeat or pulse at birth, breathing at birth, and infant movement at birth. Variables included from the VA were moving, crying, and breathing of the baby at birth. The case type (as either neonatal death or stillbirth) that was initially reported by family and the case type that was determined by the DeCoDe panel were compared for discrepancies. Signs of life variables noted above, as well as Apgar scores (>0), and whether resuscitation was attempted at birth were assessed for cases classified as stillbirths that were initially reported as neonatal deaths.

#### Sub-analysis: Resuscitation at facilities

To further characterize opportunities to use CHAMPS data to suggest measures for perinatal mortality prevention, documentation on resuscitation measures at delivery was analyzed for a subset of stillbirth cases that had completed the determining cause of death panel-review process. Resuscitation attempts at delivery as documented in the maternal health record were analyzed in stillbirth cases that were delivered at a health facility, weighed ≥ 1000 grams, were enrolled in MITS and were documented during the MITS procedure as fresh stillbirths, (i.e. those without skin sloughing or maceration, features indicating in utero pre-partum demise).

## Results

### Description of perinatal mortality cases in CHAMPS

As of January 27, 2021, 4049 neonatal deaths or stillbirths were enrolled in CHAMPS. Of these, 3641 (90%) were deaths that occurred in the perinatal period included in this analysis (Table 1). The case type classifications of perinatal deaths as reported at enrollment were: 1967 stillbirths (54%), 845 (23%) very early neonatal deaths (<24 hours old), and 829 (23%) early neonatal deaths (1 to <7 days old). Sex was listed as male in 2029 (56%), female in 1587 (44%), unknown in 20 (<1%) and indeterminate in 5 (<1%). The average ages at death were 7 hours for very early neonatal deaths and 55 hours for early neonatal deaths. Of the 3641 perinatal cases, 1822 (50%) were consented for MITS, and data recorded at the MITS procedure were available for 1725 (47%) at the time of analysis. The location of death was reported as in the community for 484 (13%), at a facility for 3145 (86%), other for 8 (<1%) and unavailable for 4 (<1%). The birth was documented as attended by a doctor, clinical officer, medical officer or assistant medical officer in 584 (16%), a midwife in 554 (15%), a nurse in 383 (11%), a specialist doctor in 215 (6%), a traditional birth attendant in 122 (3%), other in 35 (<1%), and the birth attendant was unknown or unavailable in 1748 (48%).

**Table 1:** Characteristics of perinatal mortality cases in the Child Health and Mortality Prevention Surveillance (CHAMPS) network.

	Stillbirth	Very early neonatal death	Early neonatal deaths	Total
**Total cases**	1967 (54%)	845 (23%)	829 (23%)	3641
**Gender**				
Male	1068	495	466	2029
Female	880	348	359	1587
Indeterminate	3	2		5
Unknown	16		4	20
**Average age (hours)**				
Mean	0.2	6.6	55.4	
Minimum	0.0	0.0	1.0	
Maximum	24.0	120.0	187.0	
**CHAMPS Site**				
Bangladesh	485	239	172	896
Ethiopia	134	44	37	215
Kenya	193	104	64	361
Mali	367	125	125	617
Mozambique	402	148	184	734
Sierra Leone	176	49	63	288
South Africa	210	136	184	530
**Consented for MITS**				
Yes	923	438	461	1822
No	1044	407	368	1819
**MITS procedure performed**				
Yes	870	416	439	1725
No	1097	429	390	1916
**Location of death**				
Community	208	150	126	484
Facility	1753	692	700	3145
Unknown	2	2		4
Other	4	1	3	8
**Birth attendant as documented on the maternal health record**				
Specialist Doctor	146	41	28	215
Doctor, medical or clinical officer	350	129	105	584
Nurse	218	91	74	383
Midwife	313	122	119	554
Traditional birth attendant	59	39	24	122
Other	21	9	5	35
Unknown or unavailable	140	58	58	256
No data	720	356	416	1492

### Availability of data about signs of life at birth from clinical abstractions and verbal autopsy

**Fig 1:**
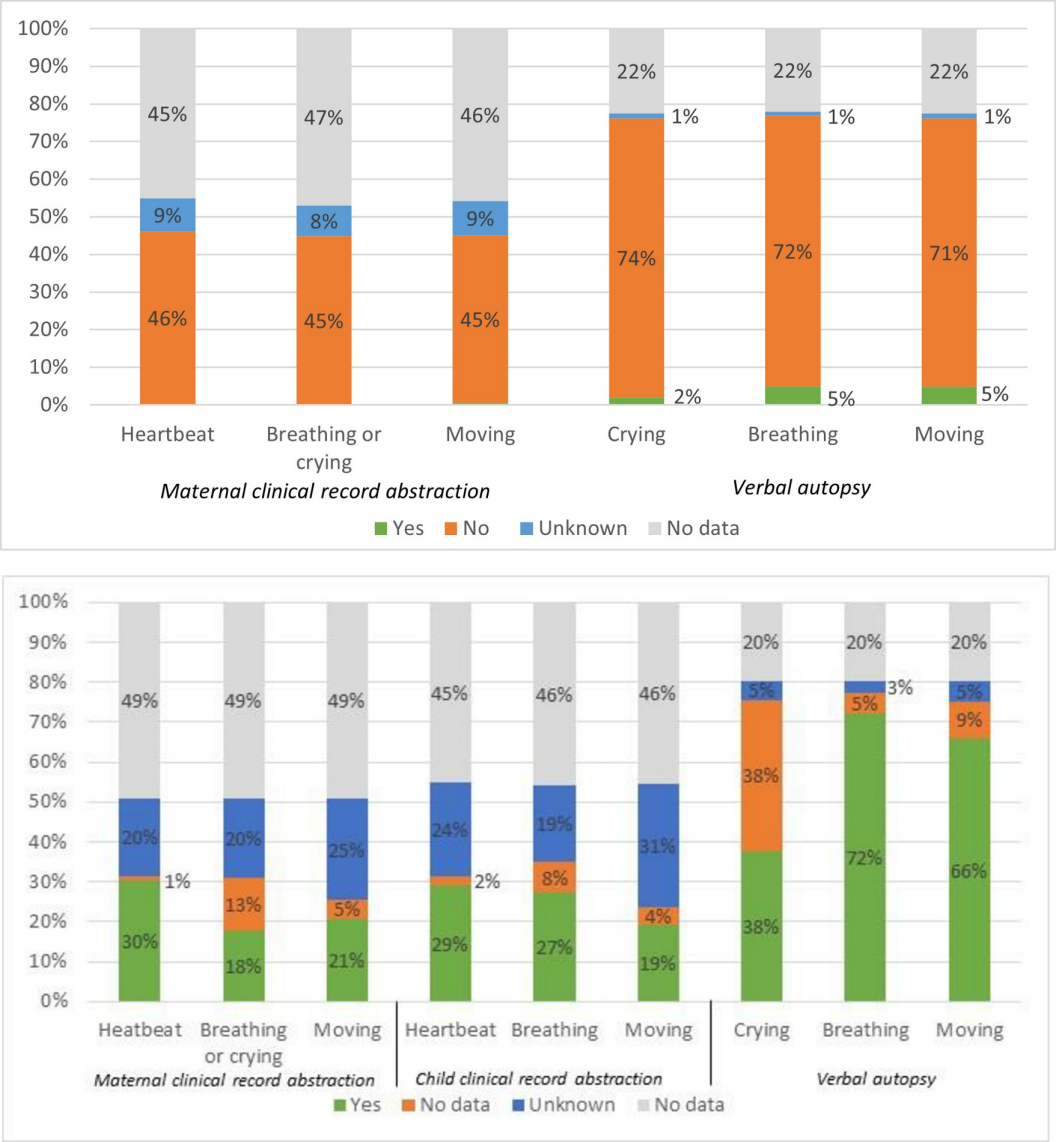
Availability and indication of signs of life from clinical health record abstractions and the verbal autopsy for stillbirths and very early neonatal deaths at facilities in the Child Health and Mortality Prevention Surveillance (CHAMPS) network. a. Stillbirths delivered in a facility (*N* = 1753). b. Very early neonatal deaths at a facility (*N* = 692).

Of stillbirths and deaths in <24 hours enrolled in CHAMPS, 1753 and 692, respectively, were born in a facility where data on signs of life at birth could have been obtained at delivery. Fig 1 shows signs of life data availability and a comparison of the clinical record and the VA data for these cases. Data were more available from the VA than from the maternal or child clinical record. For stillbirths, the availability of data on signs of life at delivery varied from 77% for breathing at birth as reported by the family on the VA to 41% on breathing and crying at birth as abstracted from the maternal clinical record (Fig 1A). While the presence of signs of life for stillbirths was reported rarely in the maternal clinical record abstraction (<1% heartbeat at birth, <1% breathing or crying at birth, and 1% movement at birth), they were reported more commonly by families on the VA (2% the baby cried, 5% the baby breathed, and 5% the baby moved).

For very early neonatal deaths in <24 hours, the availability of data on signs of life at birth varied from up to 77% for breathing at birth as reported by families on the VA to as low as 23% for movement at birth as abstracted from the neonate’s clinical record (Fig 1B). The report of families from the VA generally agreed with the assigned case type, as 72% of families reported no breathing at birth for stillbirths, and the same percentage reported breathing at birth for very early neonatal deaths.

Data availability was poor overall. Data from the maternal and child clinical record abstractions was unavailable (no data) for 45–49% and unknown or unavailable (indicated as not present in the clinical record) for an additional 19–31% of the variables on signs of life at birth in very early neonatal deaths. In stillbirths, the maternal record abstraction had no data for roughly the same proportion of cases (45–49%) across variables, with slightly fewer cases documented as unknown or unavailable (8–9%) in the maternal clinical record abstraction.

### Stillbirth and neonatal death classification

Of the 1967 cases classified as stillbirth at final case classification, 1938 (almost 99%) were initially reported as stillbirths, only 28 (1%) were reported as livebirths, and 1 (<1%) was reported as other (Table 2). Of 845 cases classified as very early neonatal death at final case classification, only 33 (4%) were initially reported as stillbirth. Of these, 32 (97%) were reclassified based on the documentation of the outcome of delivery as livebirth in the maternal health record with one additional case reclassified at the time of DeCoDe expert panel review.

**Table 2:** Concordance between stillbirths and very early neonatal deaths case final classification and reported case type at enrollment the Child Health and Mortality Prevention Surveillance (CHAMPS) network.

	Stillbirth	Livebirth	Other
Stillbirths (*N =* 1967)	n (%)	n (%)	n (%)
Final case classification	1958 (99.5%)	9 (4.5%)	0 (0%)
Reported case type at enrollment	1938 (98.5%)	28 (14.2%)	1 (0.1%)
**Very early neonatal deaths (*N* = 845)**			
Final case classification	1 (0.1%)	844 (99.8%)	0 (0.0%)
Reported case type at enrollment	33 (3.9%)	812 (96.1%)	0 (0.0%)

Of the 28 stillbirth cases initially reported as livebirths, twenty cases (71%) were reclassified based on data from the abstraction from the maternal health record that at the time of delivery the birth was documented as a stillbirth. The other 9 (32%) of these cases were reclassified based on the DeCoDe expert panel review of all available maternal health record, child health record, and VA data, including data on signs of life. None of these cases had signs of life (heartbeat, breathing or crying, or moving at birth) documented in the maternal health record (Table 3). Family members reported signs of life in 20 (71%) of these cases, with breathing at birth reported the most frequently in 19 (67%). Family members of 5 (18%) cases reported all three signs of life assessed (breathing, crying, and moving at birth), 12 (43%) reported 2 signs of life, and 3 (11%) reported 1 sign of life.

**Table 3:** Signs of life from the maternal health record, verbal autopsy, and child health records for cases reclassified after initial enrollment in the Child Health and Mortality Prevention Surveillance (CHAMPS) network.

	Very early neonatal death cases initially reported as stillbirths N = 33				Stillbirths initially reported as livebirths N = 28			
Signs of life indicated	Yes	No	Unknown	No data	Yes	No	Unknown	No data
**Maternal Health Record**								
Heartbeat	16 (48%)	2 (6%)	12 (36%)	3 (9%)	0 (0%)	19 (68%)	8 (29%)	1 (4%)
Breathing or crying	8 (24%)	8 (24%)	14 (42%)	3 (9%)	0 (0%)	21 (75%)	6 (21%)	1 (4%)
Movement	9 (27%)	5 (15%)	16 (48%)	3 (9%)	0 (0%)	19 (68%)	6 (21%)	3 (11%)
APGAR>0	27 (82%)	1 (3%)	0 (0%)	5 (15%)	1 (4%)	16 (57%)	0 (0%)	11 (39%)
**Verbal Autopsy**								
Crying	7 (21%)	17 (52%)	1 (3%)	8 (24%)	6 (21%)	15 (54%)	1 (4%)	6 (21%)
Breathing	15 (45%)	9 (27%)	1 (3%)	8 (24%)	19 (68%)	2 (7%)	1 (4%)	6 (21%)
Moving	12 (36%)	10 (30%)	3 (9%)	8 (24%)	17 (61%)	5 (18%)	0 (0%)	6 (21%)
**Child Health Record**								
Heartbeat	9 (27%)	1 (3%)	10 (30%)	13 (39%)				
Breathing	10 (30%)	2 (6%)	6 (18%)	15 (45%)				
Moving	4 (12%)	2 (6%)	14 (42%)	13 (39%)				

Of the 33 cases initially reported as stillbirth and reclassified to very early neonatal death, Apgar scores were available and >0 at 1 minute for 27 (82%), and heartbeat was present in 16 (48%) per the abstraction of the maternal health record. On the VA, family members reported breathing in 15 (45%) and moving in 12 (36%) of these cases initially classified as stillbirths.

### Resuscitation at delivery for fresh stillbirths born in a healthcare facility

Of the 1967 cases classified as stillbirth, MITS data were available for 870 (44%) at the time of this analysis. Of these cases with MITS data available, 367 (42%) were classified as fresh rather than macerated, weighed >1000g, were delivered in a facility and did not have sonographic confirmation of stillbirth prior to delivery. Abstraction of the maternal clinical record documented that resuscitation was not performed in 161 (44%), performed in 14 (3%), and unknown in 67 (18%) with data unavailable for 125 (34%). All 14 of the cases resuscitated were attended by a doctor, clinical officer, nurse, midwife, or other skilled attendant. However, in 147 (91%) of cases when the stillbirth was not resuscitated, these categories of skilled health professionals also attended the delivery (Table 4).

**Table 4:** Resuscitation performed as abstracted from clinical records a subset of in the Child Health and Mortality Prevention Surveillance (CHAMPS) network (MITS-enrolled fresh stillbirths delivered at a facility and weighing >1000g without ultrasound confirmation of stillbirth prior to delivery).

Resuscitation Attempted N = 367				
Total	Yes 14 (3%)	No 161 (44%)	Unknown/unavailable 67 (18%)	No data 125 (34%)
**Birth attendant**				
Specialist Doctor	1	23	5	5
Doctor, medical or clinical officer	7	48	20	11
Nurse	4	26	27	7
Midwife	2	50	10	3
Other	0	0	1	1
Unknown or unavailable	0	11	1	5
No data	0	3	3	93

## Discussion

This analysis found that CHAMPS cases assigned as stillbirth or neonatal death after DeCoDe expert panel review were generally consistent with the initial report of the case as a stillbirth or neonatal death, with only 1% of stillbirths and 4% of very early neonatal death cases reclassified after enrollment and during expert panel DeCoDe deliberations. Family understanding of whether a baby was born alive or stillborn and family report of the presence or absence of signs of life generally reflects available data from maternal and child health records. In maternal and child record abstractions, data elements pertinent to perinatal death classification such as those on the presence or absence of signs of life and resuscitation at birth are unavailable in many CHAMPS cases to date. However, available data in this analysis of CHAMPS MITS cases also showed that in up to 48% of fresh stillborn cases where resuscitation may have been appropriate per WHO guidelines and where delivery occurred at a health care facility, resuscitation was documented as not attempted. These data suggest a gap in implementation of basic resuscitation measures at delivery per WHO recommendations and the possibility of continued misclassification of live born infants as stillbirths. While these analyses cannot assess the potential for errors in clinical record-keeping or data abstraction, this finding indicates a need for enhanced attention to appropriate resuscitation through implementation of proven initiatives like Helping Babies Breathe as part of multi-pronged efforts to combat perinatal mortality [22]. The data gap around perinatal signs of life also supports increased attention to documentation of resuscitation attempts to improve assessment of these programs.

The provision of culturally appropriate, respectful care is not possible without a thorough understanding of the social, cultural, and economic factors contributing to individual and community perceptions of stillbirth. In addition to the value of CHAMPS’ MITS procedure results, CHAMPS’ methods that consider VA, maternal health records, and child health record enable a more thorough consideration of perinatal death classification in the context of resuscitation measures and signs of life at birth than has been available previously in similar cause of death studies. While significant data gaps remain, available data show that family and clinician understanding of the presence or absence of signs of life at birth were usually, but not always, aligned. The 71% of families in the small subset of 28 cases reclassified from neonatal death to stillbirth whose report on the VA that a baby had signs of life contradicts clinical records. The cause of this discrepant perception of the perinatal outcome between families and the healthcare system is beyond the scope of this paper and likely multifactorial and different across communities. Possibilities include differences in cultural and biomedical understanding of perinatal mortality, stigmatization around perinatal outcomes, cultural understanding of motherhood, opportunities for enhanced family-provider communication at delivery, local healthcare norms, time and resource constraints, a mother’s perception of her own treatment at delivery, or other factors [12, 23]. Further site-specific research could improve understanding of these factors to ensure culturally respectful perinatal care and to optimize context-appropriate implementation of WHO best practices.

This dataset represents a snapshot in time of the ongoing data collection through the CHAMPS Network. The abstracted clinical record data used in this study are limited to what was documented in the health facility and by any error in reading and converting often hand-written and scanty records into an electronic database. While the vast majority of cases included in this analysis were delivered in facilities, clinicians attending the delivery may have performed interventions or assessments that were not documented. Still, many cases delivered in facilities were missing essential data around perinatal resuscitation and delivery events, and those delivered in communities have even less available data. Information on signs of life were not documented or not abstracted in a substantial portion of cases. We cannot exclude the possibility of systematic bias in those cases with more complete data available. Quality projects designed to train birth attendants and clinicians to accurately assess signs of life and to improve documentation of signs of life at birth support not only neonatal resuscitation practices but also better understanding of other potential mortality-prevention strategies. These efforts will support improved classification of stillbirth and neonatal death in the CHAMPS Network and mortality surveillance efforts more broadly.

Improving accuracy of stillbirth and neonatal death classification is the first step towards quantifying the burden of each perinatal outcome in order to allocate resources to prevent and address these outcomes. The ability to detect signs of life at birth is crucial for initiating potentially life-saving interventions. These analyses leverage the multi-layered CHAMPS data to suggest opportunities for specific action, such as renewed focus on initiation of resuscitation measures at the time of delivery for live-born infants and clear documentation of signs of life at birth, Apgar scores, and reasons resuscitation is not indicated if measures are not performed.
